# Editorial: Novel Platform for Antigen Delivery to Dendritic Cells for Immunotherapy

**DOI:** 10.3389/fimmu.2022.915604

**Published:** 2022-05-30

**Authors:** Maud Plantinga, Alsya J. Affandi

**Affiliations:** ^1^ Center for Translational Immunology, divisie Laboratoria, Apotheek en Biomedische Genetica (dLAB), University Medical Center Utrecht, Utrecht, Netherlands; ^2^ Amsterdam University Medical Center (UMC) location Vrije Universiteit Amsterdam, Molecular Cell Biology and Immunology, Amsterdam, Netherlands; ^3^ Cancer Center Amsterdam, Cancer Biology and Immunology, Amsterdam, Netherlands; ^4^ Amsterdam institute for Infection and Immunity, Cancer Immunology, Amsterdam, Netherlands

**Keywords:** dendritic cells, vaccine, antigen, immunotherapy, cancer, infection, allergy, autoimmunity

Dendritic cells (DCs) are a group of antigen-presenting cells (APCs) that link innate and adaptive immune systems. DCs are specialized in processing and presenting antigens to T cells and instructing the appropriate T cells responses (1). DCs express various pattern recognition receptors (PRRs) that are capable of distinguishing ‘danger signals’ from ‘safe signals’, to ensure the proper T cell responses are initiated. For example, toll-like receptors (TLRs) mediate DCs sensing of pathogenic bacteria or viruses, which initiates cascades of immune activation. Next to this, DCs also express receptors that recognize ‘self’ structure, such as the sialic acid binding immunoglobulin type lectins (Siglecs) receptors, that promote immune suppression. Additionally, DCs integrate signals from the surrounding tissue microenvironment to further tailor the required T cell responses. In a healthy situation, DCs play a crucial role in maintaining homeostasis, by activating T cells to eliminate infected or malignant cells, or by promoting regulatory T cells to prevent chronic inflammation. Impaired immunity may result in the development of cancer, and conversely, failure to dampen immune response can lead to allergic or autoimmune diseases. This profession of the DCs lends itself particularly well for therapeutic purposes (2, 3). In this Research Topic, 9 articles cover many emerging platforms that harness DCs’ potential in re-directing T cell responses for therapeutic purposes, with a wide range of potential applications from cancer and autoimmunity, to infectious diseases (summarized in **Figure 1**).

**Figure 1:**
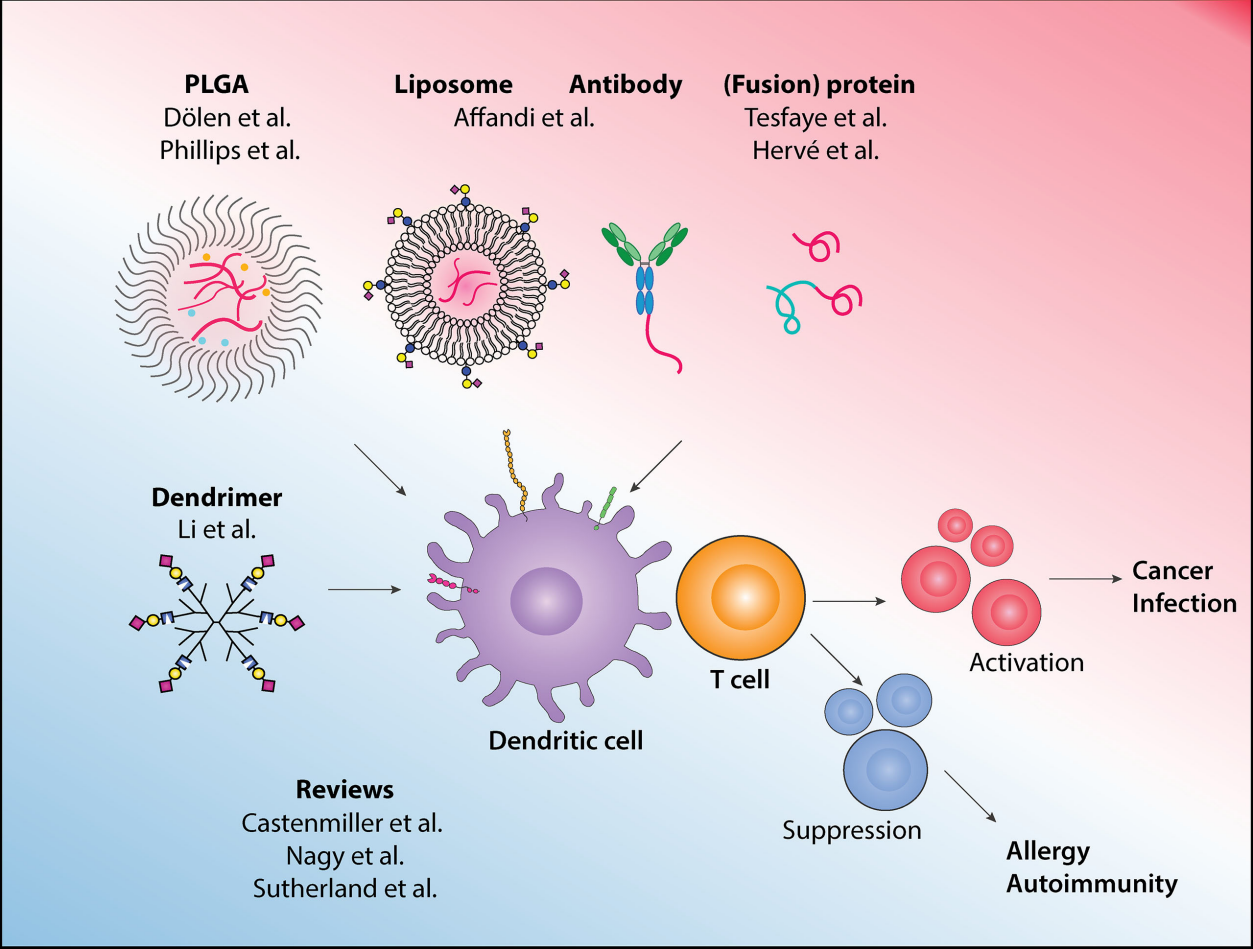
In this Research Topic, various approaches to target antigens to dendritic cells for immunotherapy and to drive the appropriate immune responses are described, and the latest development and other emerging platforms in the field are reviewed.

DCs can be broadly categorized into plasmacytoid DCs (pDCs) and conventional DCs (cDCs). While pDCs’ main function is to produce type I interferon (IFN-I), cDCs are the most potent in antigen presentation and T cell activation. cDCs can be further subdivided into distinct subsets such as the DC1 and DC2, which primarily activates CD8^+^ and CD4^+^ T cells, respectively. Furthermore, recent single-cell technologies have allowed a deeper characterization of new DC subsets that includes DC3, with hybrid CD14^+^ monocytes/DC2 phenotype, and pre-DC/AS DC (4, 5). In this collection, two reviews by Castenmiller et al. and Nagy et al. highlight recent discoveries of DC subsets, their ontogenies, and functions, as well as their potential for immunotherapies.

Due to the scarcity of DCs, initial development of DC-based therapies in cancer were prepared using enriched APCs or monocytes, which have shown promises albeit with limited clinical benefits. Focusing on prostate cancer, Sutherland et al. discuss these earlier methodologies while highlighting current advancement that allows direct isolation of blood-derived DCs, as well as *in situ* targeting technologies. Among these *in situ* targeting platforms, Dölen et al. have developed poly lactic-co-glycolic acid (PLGA)-based nanoparticles that encapsulate tumor-associated antigen NY-ESO-1 and IMM60, a novel α-GalCer analog. The inclusion of IMM60 activates invariant natural killer T (iNKT) cells, essentially serving as an adjuvant, leading to robust antigen-specific T cell and B cell responses.

To further improve antigen delivery towards DCs *in situ*, cell surface receptors that are exclusively expressed on DCs can be used as guiding molecules. In this vein, Tesfaye et al. further elaborate on their developed fusion vaccines using Xcl1 protein, a ligand for Xcr1, to deliver vaccines specifically towards DC1. This method stimulates high IgG2 production and the Xcl1-HA fusion vaccine confers protection in an influenza infection model. Affandi et al. focus on CD169, a receptor that is expressed on highly activated CD14^+^ monocytes and a small proportion of DC3. This study uses two platforms, antibody-based and liposomal-based, to deliver tumor-associated antigens to CD169-expressing CD14^+^ monocytes for effective stimulation of antigen-specific CD8^+^ T cells. Focusing on the liposome platform, Nagy et al. review how liposomes can also be used for activating or tolerizing DCs, by adjusting the physiochemical properties and the incorporated adjuvant.

Next to immune activation, this special issue also describes how DC-based therapies can be directed to establish tolerance against allergy or to suppress autoimmune responses. Castenmiller et al. discuss the most promising cell surface receptors used as targets to induce tolerogenic DCs (tolDCs), as well as various methods to target these receptors, such as antibody- or carbohydrate-antigen conjugates. In this collection, Phillips et al. formulate PLGA-microparticles that contain retinoid acid (RA) and TGFβ1. Combined with insulin autoantigen, these microparticles can target DCs and prevent disease onset in a type 1 diabetes model.

While many of the PRRs expressed by DC contain immunoreceptor tyrosine-based activation motif (ITAM) to signals for immune activation, most members of the Siglec receptor family bear immunoreceptor tyrosine-based inhibition motif (ITIM) (6). In this light, Li et al. investigate the mechanisms of how sialic acid-containing dendrimers promote tolerance using phosphoprotemic approach. This study reveals that the sialic acid/Siglec axis alters the JAK-STAT pathway on DCs and thereby promotes the immune regulating phenotype of DCs.

Finally, the route of administration also determines the type of DCs targeted, antigen routing, and the resulting immune responses (7, 8). Work by Hervé et al. describes that in sensitized animals, pre-existing antibodies enhance allergen uptake by migratory DCs upon epicutaneous application. This mainly involves IgG and IgG Fc receptors (FcγR) and this approach may also have the potential for a needle-free booster vaccination strategy.

Targeting DCs is a promising approach to harnessing a patient’s immune system. However, only through the effective delivery of antigens and adjuvants directly to DCs, the goal will be reached of vaccines that can stimulate adequate T cell responses for the treatment of diseases including cancer, infection, allergy, or autoimmunity. The articles in this issue highlight emerging technologies and describe several novel platforms that can optimize DCs’ potential for immunotherapy.

## Author Contributions

All authors listed have made a substantial, direct, and intellectual contribution to the work, and approved it for publication.

## Conflict of Interest

The authors declare that the research was conducted in the absence of any commercial or financial relationships that could be construed as a potential conflict of interest.

## Publisher’s Note

All claims expressed in this article are solely those of the authors and do not necessarily represent those of their affiliated organizations, or those of the publisher, the editors and the reviewers. Any product that may be evaluated in this article, or claim that may be made by its manufacturer, is not guaranteed or endorsed by the publisher.
